# The contribution of risk factors to socioeconomic inequalities in multimorbidity across the lifecourse: a longitudinal analysis of the Twenty-07 cohort

**DOI:** 10.1186/s12916-017-0913-6

**Published:** 2017-08-24

**Authors:** Srinivasa Vittal Katikireddi, Kathryn Skivington, Alastair H. Leyland, Kate Hunt, Stewart W Mercer

**Affiliations:** 1 MRC/CSO Social & Public Health Sciences Unit, University of Glasgow, Top floor, 200 Renfield Street, Glasgow, G2 3QB United Kingdom; 2 Department of General Practice & Primary Care, University of Glasgow, 1 Horselethill Road, Glasgow, G12 8UX Scotland

**Keywords:** Multimorbidity, Comorbidity, Health behaviour, Risk factors, Health care disparities, Socioeconomic status

## Abstract

**Background:**

Multimorbidity is a major challenge to health systems globally and disproportionately affects socioeconomically disadvantaged populations. We examined socioeconomic inequalities in developing multimorbidity across the lifecourse and investigated the contribution of five behaviour-related risk factors.

**Methods:**

The Twenty-07 study recruited participants aged approximately 15, 35, and 55 years in 1987 and followed them up over 20 years. The primary outcome was development of multimorbidity (2+ health conditions). The relationship between five different risk factors (smoking, alcohol consumption, diet, body mass index (BMI), physical activity) and the development of multimorbidity was assessed. Social patterning in the development of multimorbidity based on two measures of socioeconomic status (area-based deprivation and household income) was then determined, followed by investigation of potential mediation by the five risk factors. Multilevel logistic regression models and predictive margins were used for statistical analyses. Socioeconomic inequalities in multimorbidity were quantified using relative indices of inequality and attenuation assessed through addition of risk factors.

**Results:**

Multimorbidity prevalence increased markedly in all cohorts over the 20 years. Socioeconomic disadvantage was associated with increased risk of developing multimorbidity (most vs least deprived areas: odds ratio (OR) 1.46, 95% confidence interval (CI) 1.26–1.68), and the risk was at least as great when assessed by income (OR 1.53, 95% CI 1.25–1.87) or when defining multimorbidity as 3+ conditions. Smoking (current vs never OR 1.56, 1.36–1.78), diet (no fruit/vegetable consumption in previous week vs consumption every day OR 1.57, 95% CI 1.33–1.84), and BMI (morbidly obese vs healthy weight OR 1.88, 95% CI 1.42–2.49) were strong independent predictors of developing multimorbidity. A dose–response relationship was observed with number of risk factors and subsequent multimorbidity (3+ risk factors vs none OR 1.91, 95% CI 1.57–2.33). However, the five risk factors combined explained only 40.8% of socioeconomic inequalities in multimorbidity development.

**Conclusions:**

Preventive measures addressing known risk factors, particularly obesity and smoking, could reduce the future multimorbidity burden. However, major socioeconomic inequalities in the development of multimorbidity exist even after taking account of known risk factors. Tackling social determinants of health, including holistic health and social care, is necessary if the rising burden of multimorbidity in disadvantaged populations is to be redressed.

**Electronic supplementary material:**

The online version of this article (doi:10.1186/s12916-017-0913-6) contains supplementary material, which is available to authorized users.

## Background

Multimorbidity is one of the largest challenges facing health systems worldwide [1, 2]. Multimorbidity—usually defined as experiencing two or more chronic conditions—highlights the limitations of existing health care systems, which are often ill-suited to meet the needs of those affected. Traditionally, medical practice has focussed on diagnosing and treating single health conditions in isolation [3, 4]. Multimorbidity is common in the general population [5–8] and increases with age [5, 9], but it is not limited to the older population, since often a greater number of people affected are aged under 65 years [5, 9].

Socioeconomically disadvantaged populations experience a greater burden of multimorbidity and are affected from an earlier age [5, 7]. Prevention-based approaches, often focussing on well-established risk factors such as smoking, diet, and physical activity, are being prioritised by health policy to both improve population health and address inequalities [10, 11]. However, the importance of these risk factors for multimorbidity across the lifecourse and their contribution to socioeconomic inequalities are unknown. We aimed to describe the development and social patterning of multimorbidity over the lifecourse and quantify the contribution of behaviour-related risk factors.

## Methods

### Data source

We used data from the West of Scotland Twenty-07 cohort study (hereafter ‘Twenty-07’), which was started in 1987 to investigate social processes that produce or maintain health inequalities. Study details have been previously published [12, 13]. Twenty-07 employed a two-stage stratified random sample of respondents from three cohorts, born in the early 1930s, 1950s and 1970s (baseline approximate age 15, 35, 55 years) and residing in the west of Scotland. Comparison with census data showed that the sample was broadly representative of the area [14]. Respondents were recruited from 62 sampling units (postcode sectors, average population of around 5000 people). There were 4510 respondents at baseline, and there have been four follow-up waves: 1990–1991 (*n* = 3820); 1995–1997 (*n* = 2972); 2000–2004 (*n* = 2661); 2007–2008 (*n* = 2604). Respondents were aged approximately 35, 55, and 75 at the final data collection wave, which included 67% of the baseline sample who were still alive. All cohorts and data collection waves were used in the analysis, except for the 1970s cohort at wave 1 (aged 15), as risk factor information was not collected in a comparable way at this age. We therefore analyse data on individuals aged across the adult lifecourse, from 18 to 75 years. Ethical approval was obtained at each wave from National Health Service (NHS) and/or University of Glasgow research ethics committees. Respondents are flagged for routine follow-up for mortality through the health service registry; 674 had died prior to the final data collection in 2007/2008.

### Measures

#### Outcomes

Multimorbidity is typically characterised by the presence of two or more chronic conditions [5], but the actual conditions included vary. We defined chronic conditions on the basis of those used by Barnett and colleagues in their landmark study [5], whose work was informed by a previous systematic review of multimorbidity indices [15]. Self-reported conditions were coded based on the Royal College of General Practitioners’ Morbidity classification [16]. The exact coding process was discussed and agreed on by two clinically qualified members of the research team (SVK and SM). Respondents who had two or more of the 40 relevant conditions were classed as having multimorbidity. Further details on the diagnostic coding are provided in Additional file 1: Table S1.

#### Socioeconomic status

Area-based deprivation categories were calculated using Carstairs scores for postcode sectors. Carstairs scores provide an index of deprivation based on census results for four indicators of socioeconomic status (car ownership, male unemployment, overcrowding, and low social class) for residents of each postcode sector [17]. The seven categories from Twenty-07 were collapsed into three: least, intermediate, and most deprived. Household income was used in supplementary analyses to compare associations with an alternative measure of socioeconomic status. To make the income variable comparable across households, it was weighted for number and age of people living in the household, using the McClements equivalence scale [18]. To take account of period effects, tertiles of income were created separately for each cohort and wave.

Relative indices of inequality (RIIs) were used to investigate the potential contribution of risk factors to socioeconomic patterning of multimorbidity [19]. Participants from each cohort at each wave were ranked from lowest to highest deprivation score, with the mid-point of each deprivation category in the cumulative distribution used. These rank-based measures were then standardised to produce an index that ranged from zero (the hypothetically most disadvantaged) to one (the hypothetically most advantaged) and then regressed on the outcome, with 95% confidence intervals (CIs) obtained from estimation of the regression coefficient.

#### Risk factors

Risk factors were obtained through surveys administered using standardised protocols, with the exception of body mass index (BMI), which was nurse-measured. Diet was classified on the basis of frequency of fruit and vegetable consumption in the 7 days prior to interview into: ate fruit or vegetables every day; ate fruit or vegetables some days; had not eaten fruit or vegetables. Exercise was estimated by number of days per week of activity lasting at least 20 minutes which made the respondent out of breath or sweaty, categorised into: none, 1–3, and >3 days. For smoking status, respondents were classified into: never smoker, ex-smoker, and current smoker. BMI (weight (kg)/height (m^2^)) utilised standard thresholds: underweight (BMI <18.5), normal range (BMI 18.5–24.9), overweight (BMI 25.0–29.9), obese (BMI 30.0–34.9), and morbidly obese (BMI >34.9). Respondents were asked about alcoholic drinks consumed in the week prior to interview, and units of alcohol were calculated based on amount and type. Two measures of alcohol intake were created: exceeding existing weekly recommended maximum guidelines, and exceeding daily recommended maximum guidelines (binge drinking) in the previous week. Males exceeded weekly guidelines if they consumed more than 21 units of alcohol a week, and exceeded binge drinking guidelines if they consumed more than 10 units in one session. Equivalent figures for females were 14 and 7 respectively. Daily units of alcohol intake were not available for all cohorts at all waves and were therefore used only in supplementary analyses. Lastly, a count score was created by adding up the number of adverse risk factors.

### Statistical analyses

The data structure was hierarchical. Multilevel logistic regression models were used to assess the relationship between multimorbidity and potential socioeconomic and health-related risk factors. Models were constructed in Stata version 13 using three levels: measurement points (*n* = 9277), within individuals (*n* = 3466), and within sampling units (*n* = 62), estimated using the adaptive Gauss-Hermite quadrature option in the melogit command.

To understand how multimorbidity prevalence has changed over time, we modelled prevalence across the lifecourse by predicting the probability of having multimorbidity using our main statistical model described below. Modelled rather than crude prevalence was assessed to account for potential bias arising from attrition.

For the main longitudinal analysis, the outcome at each wave was modelled based on deprivation and risk factor predictors from the previous wave, effectively modelling change over time. For example, multimorbidity outcomes at wave 5 were modelled using deprivation and risk factor predictors measured at wave 4. The first set of models examined multimorbidity at waves 2–5 (waves 3–5 for the 1970s cohort) with explanatory variables age, sex, cohort, and multimorbidity at the previous wave (waves 1–4, and waves 2–4 for the 1970s cohort). In the second set of models, deprivation and each risk factor were added, separately, to Model 1 (Model 1a–g). Risk factors were then added jointly to determine whether they showed independent associations with multimorbidity (Model 2).

All models were adjusted for age, sex, cohort, time between waves, and multimorbidity at the previous data collection point. Odds ratios (ORs) and 95% CIs were obtained. The modelling strategy meant that each wave was conditional on data being available at the previous wave, hence outcomes at baseline (wave 1 for the 1950s and 1930s cohort and waves 1 and 2 for the 1970s cohort) were not modelled. Each participant could be included in the model up to four times, if they participated in all five waves (and up to three times for the 1970s cohort).

Interactions between cohort, sex, and age, and with all risk factor and socioeconomic status variables were tested using the global Wald test; final models included significant interactions. There were no significant interactions between risk factors and age, sex, or cohort (except for income and binge drinking, used in supplementary analyses). However, interactions between sex and cohort were statistically significant and are included in all analyses. The best fitting function of age was cubic, which was included in the model in addition to linear and quadratic age terms.

To illustrate the results, we present predicted probabilities for developing multimorbidity across the lifecourse. Stata’s margins command was used, and for these graphs only, the sample was restricted to those who were not multimorbid at the prior wave and the fixed part of the regression models used for prediction. Separate curves were drawn for each covariate. Lastly, we investigated how much each risk factor mediated socioeconomic inequalities in multimorbidity by assessing the percentage change in the coefficient for deprivation after adding each risk factor, using the approach of Stringhini et al. [20, 21], i.e. comparing the coefficients for the magnitude of inequalities across different regression models.

To minimise potential bias arising from missing data (see Additional file 1: Table S6 for details on missing data), we used multiple imputation with chained equations to address both item and wave missingness. Imputed data for covariates were not used when there was attrition from the study (i.e. data were imputed when participants temporarily did not engage with the study but not when no further survey participation occurred). Imputed outcome data were used when covariates were available at the previous wave. We included auxiliary variables that were correlated with the outcome (social class and self-rated health) when imputing data. We conducted 24 rounds of multiple imputation. The regression models and the calculation of relative indices of inequality are based on the multiply imputed data; a complete case analysis yielded similar findings (see Additional file 1: Table S10). Given that there were similar findings between imputed and complete case data, predicted probabilities were calculated using complete case data.

We conducted supplementary analyses to check the robustness of our findings. As results were similar using complete case and imputed data, we used complete case data for the supplementary analyses. To further explore alcohol intake, guidance on daily recommended units was used to calculate binge drinking, and this was added as a risk factor. As a different measure of socioeconomic status, the associations with household income were compared to those of area-based deprivation. Given the potential for death introducing survivorship bias, experiencing death or multimorbidity was defined as a secondary outcome in sensitivity analyses. A further definition of the outcome, multimorbidity as three or more conditions, was also used. We also implemented an alternative mediation modelling approach using Stata’s khb command, which overcomes some methodological concerns about traditional approaches to mediation analysis [22]—in particular, the limitation that regression coefficients from different logistic regression models should not be compared.

## Results

### Prevalence of multimorbidity

**Fig. 1:**
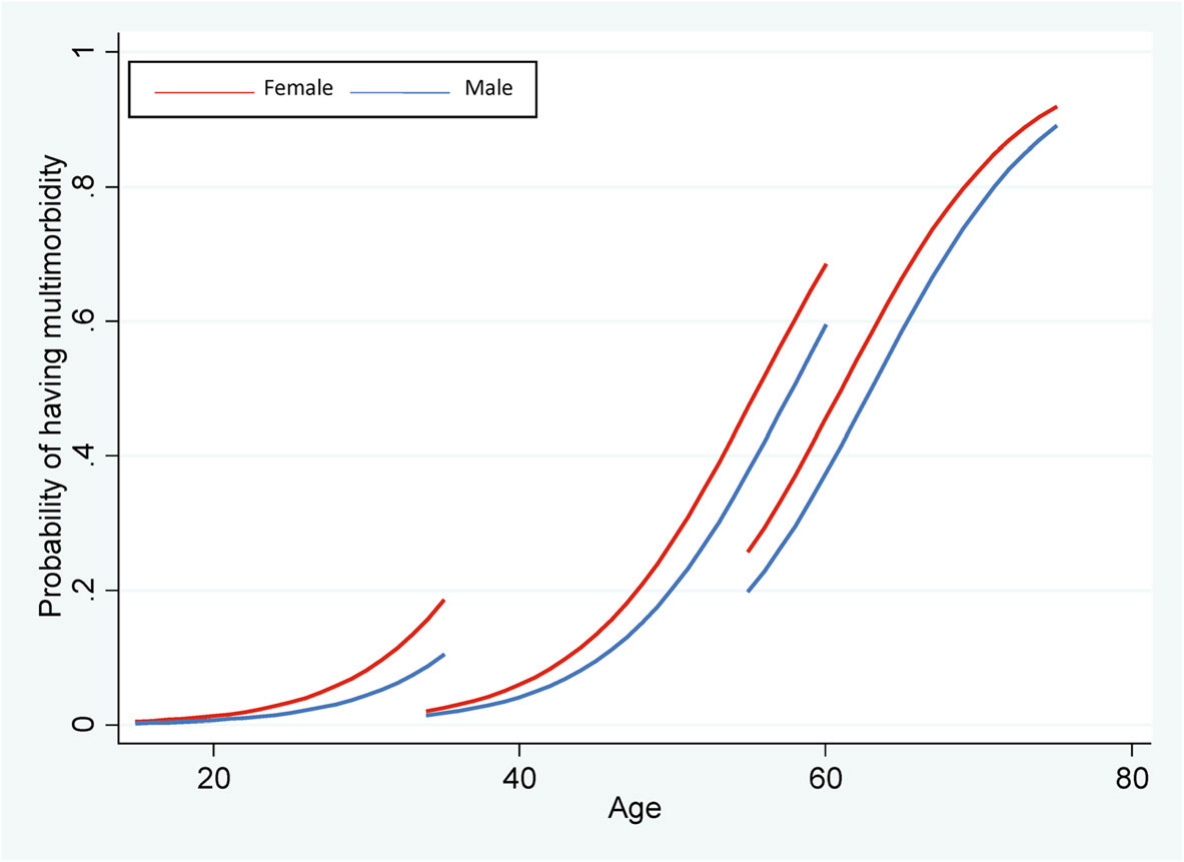
Predicted prevalence of multimorbidity across the lifecourse by cohort and sex

Baseline prevalence of multimorbidity differed substantially by age (2.6% at 19 years, 4.2% at 35 years, and 35.0% at 55 years) (Additional file 1: Table S2). Multimorbidity prevalence increased substantially over the 20-year period in all age groups. Figure 1 shows the predicted prevalence of multimorbidity over the lifecourse for each cohort, accounting for attrition. Younger cohorts experienced higher prevalence of multimorbidity when at the same age as older cohorts. For example, men born in the 1950s experienced a 59% higher prevalence of multimorbidity at the age of 60 years, compared to men born in the 1930s (59% vs 37% prevalence). At baseline, the most prevalent health conditions were pain, depression, hypertension, respiratory conditions, and dyspepsia (Additional file 1: Table S3). As expected, there were large differences by cohort, with the most common conditions in the youngest cohort being depression, anxiety, and respiratory conditions (Additional file 1: Table S4).

### Predictors of developing multimorbidity

Females had higher levels of multimorbidity in all three cohorts at baseline and throughout the follow-up period. An interaction between gender and cohort indicated that gender differences in the development of multimorbidity increased in the 1970s and 1950s cohorts compared to the 1930s cohort (multimorbidity for females from the 1970s cohort relative to the 1930s cohort: OR 1.51; 95% CI 1.11–2.05). The null models and interaction effects are shown in Additional file 1: Table S5.

When added to the regression model separately, deprivation and all risk factors, except for physical activity, were significant predictors of multimorbidity (see Table 1; Model 1). The more risk factors present within an individual, the higher the odds of developing multimorbidity; those with three or more risk factors had almost double the odds (OR 1.91; 95% CI 1.57–2.33) of developing multimorbidity compared to those with no risk factors.

**Table 1:** Predictors of developing multimorbidity in the Twenty-07 study

Independent variables		Model 1a–g: separate models for each risk factor plus deprivation		Model 2: mutually adjusted for all risk factors	
		OR	(95% CI)	OR	(95% CI)
Area-based deprivation	Least	1			
	Intermediate	1.28	(1.12–1.47)*		
	Most	1.46	(1.26–1.68)*		
Smoking	Never	1		1	
	Ex	1.33	(1.16–1.53)*	1.35	(1.18–1.55)*
	Current	1.56	(1.36–1.78)*	1.57	(1.37–1.80)*
Alcohol units (recommended weekly units)	No excess	1		1	
	Exceeds	1.07	(0.94–1.23)	1.01	(0.88–1.15)
	None/ex	1.50	(1.27–1.77)*	1.49	(1.26–1.76)*
Diet (fruit or vegetable consumption)	Everyday	1		1	
	Some days	1.10	(0.99–1.23)	1.06	(0.95–1.19)
	No days	1.57	(1.33–1.84)*	1.45	(1.24–1.71)*
Physical activity	3+ days	1		1	
	1–3 days	0.94	(0.81–1.09)	0.92	(0.79–1.08)
	None	1.02	(0.89–1.16)	0.97	(0.85–1.10)
BMI	Healthy	1		1	
	Overweight	1.21	(1.08–1.36)*	1.26	(1.12–1.41)*
	Obese	1.37	(1.17–1.61)*	1.43	(1.21–1.68)*
	Morbidly obese	1.88	(1.42–2.49)*	1.98	(1.50–2.62)*
	Underweight	1.24	(0.82–12.87)	1.13	(0.74–1.73)
Risk factor count	0	1			
	1	1.22	(1.04–1.44)*		
	2	1.51	(1.28–1.79)*		
	≥3	1.91	(1.57–2.33)*		

All models are adjusted for age, age squared, age cubed, sex, cohort, prior multimorbidity, time between waves and sex*cohort interaction. Based on 24 multiply imputed datasets

*p < 0.05

**Fig. 2:**
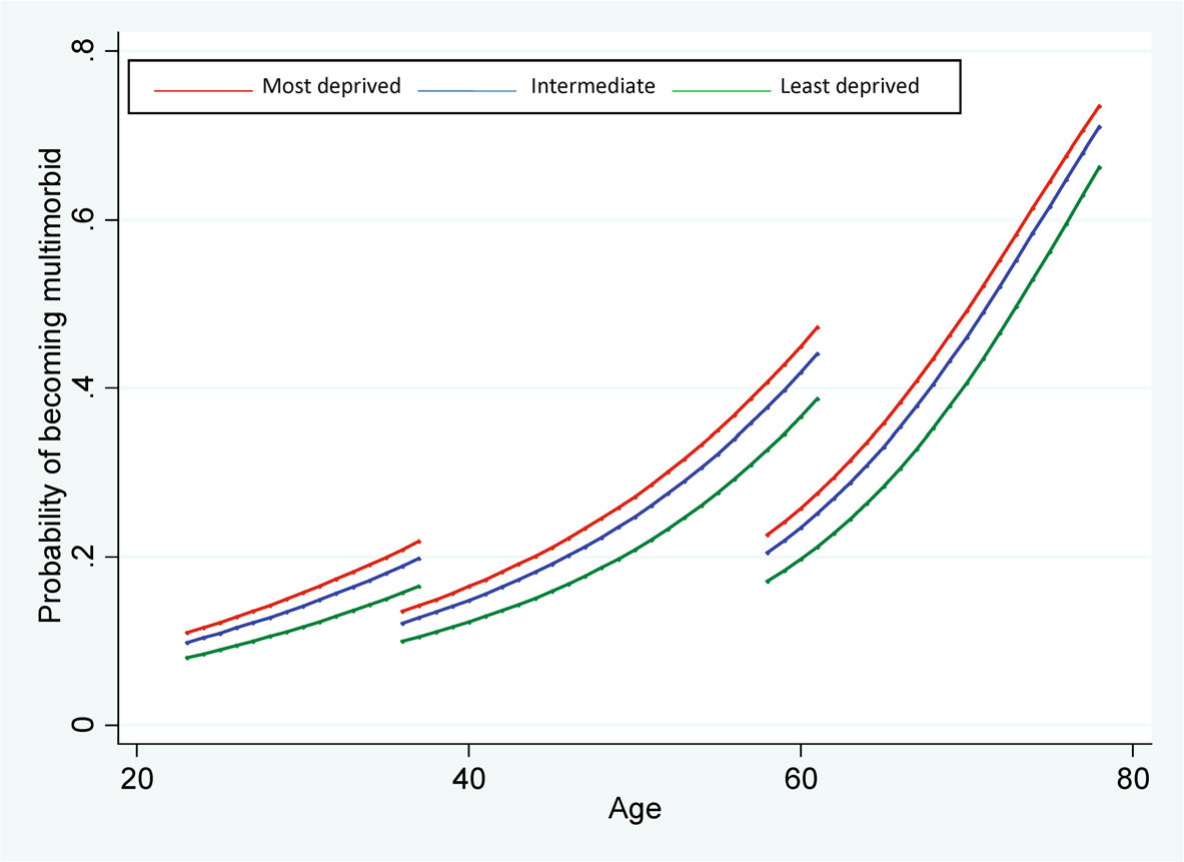
The independent contribution of area deprivation to the predicted probability of developing multimorbidity over a 5-year period in the Twenty-07 study (Adjusted for age, age squared, age cubed, sex, cohort, previous multimorbidity, time between waves, and cohort*sex interaction. The above does not include risk factors within the statistical model)

Figure 2 shows the relationship between deprivation and developing multimorbidity; those in the least deprived areas had a lower predicted probability of developing multimorbidity than those in the most deprived areas. The inequalities gap was most apparent between 50 and 70 years (approximately 8.0% absolute difference in the risk of developing multimorbidity over a 5-year time period at 55 years of age), but was found in younger and older adulthood as well.

**Fig. 3:**
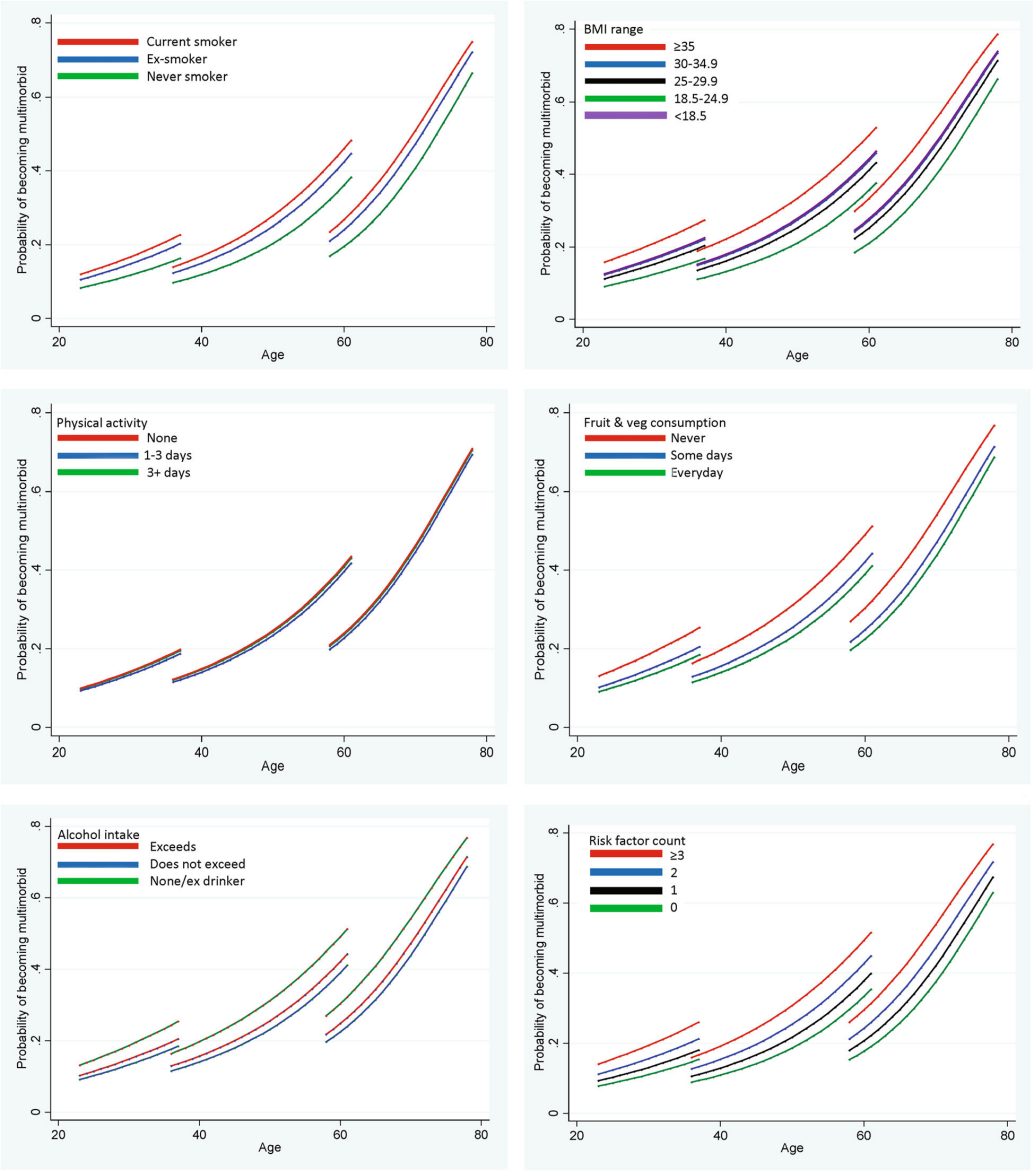
The independent contribution of risk factors to the predicted probability of developing multimorbidity over a 5-year period in the Twenty-07 study (Adjusted for age, age squared, age cubed, sex, cohort, previous multimorbidity, time between waves, and cohort*sex interaction)

Figure 3 shows age trajectories of developing multimorbidity by smoking, BMI, physical activity, diet, alcohol, and risk factor count. There were clear differences in probability of developing multimorbidity for those who were overweight, current or ex-smokers, those with poor diet, and those reporting no alcohol consumption. A similar pattern was apparent when investigating the risk factor count. As with deprivation, absolute differences between multimorbidity for each BMI, diet, alcohol, and smoking category were most apparent in later mid-life, with smaller absolute differences seen in young and older adulthood. For example, at age 59, current smokers had a 30% predicted probability of developing multimorbidity over 5 years compared to 22% for never smokers; whereas at age 23, the respective probabilities were 11% and 7%.

Lastly, the extent to which the five risk factors attenuated the relationship between area-based deprivation and multimorbidity was investigated by comparing the magnitude of inequalities before and after adjusting for different risk factors (Table 2). The RII which summarises the extent of inequality in the development of multimorbidity was 1.74 (95% CI 1.44–2.11). There was modest attenuation of inequalities when adding risk factors to the regression models, with smoking (15.4%) followed by diet (11.9%) making the greatest contribution to inequalities. Accounting for differences in all five risk factors resulted in a total of 40.8% attenuation, indicating that the majority of inequalities in the development of multimorbidity remained unexplained by these five risk factors.

**Table 2:** Relative indices of inequality (RII) in the development of multimorbidity, calculated by area-level deprivation

	RII odds ratio (95% CI)^a^	% attenuation^b^
Null model	1.74 (1.44–2.11)	NA
Plus smoking	1.60 (1.32–1.94)	15.4
Plus diet	1.63 (1.35–1.97)	11.9
Plus physical activity	1.73 (1.43–2.09)	1.1
Plus alcohol	1.69 (1.40–2.05)	5.3
Plus BMI	1.67 (1.38–2.01)	8.3
Plus all five risk factors	1.39 (1.15–1.68)	40.8
Plus risk factor count	1.58 (1.31–1.90)	17.8

^a^Adjusted for prior multimorbidity, age, age squared, age cubed, sex, cohort, time between waves, and sex*cohort interaction. Based on 24 multiply imputed datasets

^b^Calculated using the approach of Stringhini et al. [20]: 100*(βModel 2a – βModel 2a + risk factor)/βModel 2a

Supplementary analyses with household income instead of area deprivation (Additional file 1: Table S7), daily alcohol (binge drinking) rather than weekly alcohol units (Additional file 1: Table S8), and different definitions of multimorbidity showed largely similar patterns of findings. Inequalities by income were slightly greater than by deprivation, and the risk of developing multimorbidity was clearly increased amongst binge drinkers in contrast to the greatest risk amongst non-drinkers within the main analysis. Using a combined outcome of multimorbidity or mortality showed almost identical findings as those seen in the main analysis, suggesting that survivorship bias did not account for the findings (Additional file 1: Table S9). Limiting the main analysis to complete data also yielded similar findings (Additional file 1: Table S10). Risk factors were stronger predictors of multimorbidity when it was defined as being three or more conditions rather than two (Additional file 1: Table S11). For example, in the most deprived group, the OR for developing multimorbidity was 2.04 (95% CI 1.46–2.84) for 3+ chronic conditions compared to 1.46 (95% CI 1.26–1.68) when 2+ conditions were used. With multimorbidity defined as three or more conditions, the RII was 2.52 (95% CI 1.78–3.57) in the null model, with 33.7% attenuation when adjusting for all risk factors (Additional file 1: Table S12). Lastly, using the alternative statistical approach, the Karlson, Holm, and Breen (KHB) method, to investigate the mediation of five behaviour-related risk factors on socioeconomic inequalities in multimorbidity led to similar results (Additional file 1: Table S13).

## Discussion

Socioeconomic status predicts the development of multimorbidity throughout the adult lifecourse, with inequalities greatest between 50 and 70 years. Modifiable risk factors (such as smoking, lack of physical activity, and poor diet) for several chronic conditions are important predictors of developing multimorbidity. While these risk factors appear to partially mediate the relationship between deprivation and multimorbidity, the majority of the relationship between deprivation and developing multimorbidity remains unexplained.

Efforts to reduce these known risk factors at any point across the lifecourse could contribute to addressing the growing burden of multimorbidity, with the greatest potential impact when changing behaviour before age 50. However, behaviour-related risk factors only mediate the association between deprivation and multimorbidity to a limited extent; most of this association is unexplained. It is essential to also focus attention on addressing the underlying causes of deprivation to reduce health inequalities [23]. The most socioeconomically disadvantaged experience the greatest burden of multimorbidity, and in addition to the imperative to tackle such inequality, health systems also need to be responsive to it. Effective health care that is based on a collaborative, comprehensive, patient-centred system is required to deal with such patient complexity [24–26]. A starting point is universal coverage of health care, especially primary care. However, even in such systems, the inverse care law persists, affecting patients of low socioeconomic status with complex multimorbidity the most [27, 28].

Our study has several important strengths. We investigate a well-characterised longitudinal cohort designed to cover the entire adult lifecourse. Furthermore, the sample is representative of the general population, and we collected social variables in a standardised manner. However, some limitations should be noted. First, multimorbidity was defined on the basis of self-reported health conditions, which may lead to bias in outcome ascertainment [29]. However, health conditions were coded using standardised criteria by trained health professionals. Second, most risk factor information was self-reported—likely to be particularly problematic for physical activity. A related issue is that the measures capture behaviour at a particular time point, with reverse causation likely to explain the findings related to alcohol. In addition, the measures of diet and physical activity only captured specific aspects of these behaviour-related risk factors. Aspects of diet, other than fruit and vegetable consumption, which may be associated with development of multimorbidity (e.g. salt and saturated fat consumption) could not be included. Likewise, further details on physical activity (e.g. type of activity, whether it was work or leisure related, and more information on how much activity) could not be included. Third, there are other potentially important dimensions of socioeconomic position which we have not been able to investigate; future investigation may provide a greater level of understanding and afford opportunities for intervention. For example, low education has been linked to multimorbidity [30] and is also closely linked to health literacy, thereby highlighting a potential target for intervening to influence health behaviour. Fourth, all cohorts experience attrition, although given the diverse socioeconomic population studied in the Twenty-07 study, this is relatively modest. Fifth, our variables have some missing data, which we addressed through multiple imputation.

## Conclusions

Our study demonstrates the existence of substantial socioeconomic inequalities in the development of multimorbidity throughout the adult lifecourse. While common risk factors predispose to the development of multimorbidity, a key finding of this study is that the relationship between deprivation and the development of multimorbidity is only partially mediated by lifestyle factors. Future research is required to identify other factors that mediate the relationship between deprivation and multimorbidity. Furthermore, there is a need for further research to distinguish the relative importance of mental and physical health conditions [31] as well as investigate combinations of specific health conditions in greater detail [32, 33].

Irrespective of the biological pathways, policies and interventions focussing on the social determinants of health may be the key to reducing the prevalence and severity of multimorbidity. Social determinants include access to high-quality health care, with the continuing existence of the ‘inverse care law’ likely contributing to excess multimorbidity in deprived areas. A recent exploratory cluster randomised controlled trial which supported general practitioners and practice nurses working in deprived areas to deliver holistic, person-centred care to patients with multimorbidity led to cost-effective improvement in wellbeing [34, 35]. Beyond this, a focus on addressing non-health sector determinants, including income, employment, housing, and the social and physical environment, is necessary [36–38].

## Additional file

Additional file 1: Supplementary analyses.** Table S1. ** Classification of multimorbidity. **Table S2.** Descriptive characteristics of study participants. **Table S3.** Number (*n*) and proportion of sample with each condition, by wave. **Table S4.** Proportion of sample with each condition by cohort at baseline. **Table S5.** Tests of statistical interaction. **Table S6.** Summary of missing values for imputed variables. **Table S7.** Comparisons of area deprivation and income. **Table S8.** Comparisons of weekly alcohol consumption and binge drinking. **Table S9.** Odds of multimorbidity or death. **Table S10.** Complete case analysis. **Table S11.** Odds of multimorbidity based on 3+ conditions. **Table S12.** Relative indices of inequality based on 3+ conditions. **Table S13.** Relative indices of inequality according to Karlson, Holm, and Breen (*KHB*) method. (DOC 492 kb)

## Abbreviations

95% CI: 95% Confidence interval
BMI: Body mass index
kg: Kilogramme
m: Metre
*n*: Number within sample
OR: Odds ratio
RII: Relative index of inequality
