# Supplementary material for: The contribution of risk factors to socioeconomic inequalities in multimorbidity across the lifecourse: a longitudinal analysis of the Twenty-07 cohort
Source: BMC Med. 2017 Aug 24;15:152. doi: 10.1186/s12916-017-0913-6 (PMC5569487; doi:10.1186/s12916-017-0913-6)
Supplement: Additional file 1: — Supplementary analyses. Table S1. Classification of multimorbidity. Table S2. Descriptive characteristics of study participants. Table S3. Number (n) and proportion of sample with each condition, by wave. Table S4. Proportion of sample with each condition by cohort at baseline. Table S5. Tests of statistical interaction. Table S6. Summary of missing values for imputed variables. Table S7. Comparisons of area deprivation and income. Table S8. Comparisons of weekly alcohol consumption and binge drinking. Table S9. Odds of multimorbidity or death. Table S10. Complete case analysis. Table S11. Odds of multimorbidity based on 3+ conditions. Table S12. Relative indices of inequality based on 3+ conditions. Table S13. Relative indices of inequality according to Karlson, Holm, and Breen (KHB) method. (DOC 492 kb) [file 12916_2017_913_MOESM1_ESM.doc]

# Additional File

Contents

Table S1: Classification of multimorbidity

Table S2: Descriptive characteristics of study participants

Table S3: Number (n) and proportion of sample with each condition, by wave

Table S4: Proportion of sample with each condition by cohort at baseline

Table S5: Tests of statistical interaction

Table S6: Summary of missing values for imputed variables

Table S7: Comparisons of area deprivation and income

Table S8: Comparisons of weekly alcohol consumption and binge drinking

Table S9: Odds of multimorbidity or death

Table S10: Complete case analysis

Table S11: Odds of multimorbidity based on 3+ conditions

Table S12: Relative indices of inequality based on 3+ conditions

Table S13: Relative indices of inequality according to KHB method

**Classification of multimorbidity**

The data cleaning step for conditions across the study period involved cross-checking with all other available information on medications, other conditions, and verbatim responses to determine the most appropriate response for each participant at each wave. After any errors were resolved we then created rules for those conditions that were judged (by SVK and SM, who have clinical expertise) to be life-long (marked with *).

Table S- : Coding of multimorbidity on the basis of the Royal College of General Practitioners’ 1986 classification system

| **CONDITION** | **CODE** |
| --- | --- |
| **HYPERTENSION** | 1910* |
|  | 1915* |
|  | 1920* |
|  | 1925* |
|  | 1935* |
| **DEPRESSION** | 1060 |
| **RESPIRATORY CONDITIONS including asthma** | 2500 |
|  | 2510 |
|  | 2490* |
|  | 2495* |
| **CORONARY HEART DISEASE** | 1940* |
|  | 1945* |
|  | 1950* |
|  | 7735* |
| **DYSPEPSIA** | 2660 |
|  | 2675 |
|  | 2680 |
|  | 2685 |
|  | 2690 |
|  | 2695 |
|  | 2700 |
|  | 4660 |
| **DIABETES** | 0720* |
| **THYROID** | 0700 |
|  | 0705* |
|  | 0710* |
|  | 0715* |
|  | 7570* |
| **RHEUMATOID ARTHRITIS, other inflammatory polyarthropathies & systematic connective tissue disorders** | 3905* |
|  | 3910* |
|  | 4510* |
|  | 0770 |
|  | 3690* |
|  | 3900* |
| **HEARING PROBLEMS** | 1740 |
|  | 1745 |
|  | 1750 |
|  | 1770 |
|  | 1775 |
|  | 1780 |
| **ANXIETY & OTHER NEUROTIC, STRESS-RELATED, & SOMATOFORM DISORDERS** | 1040 |
|  | 1045 |
|  | 1050 |
|  | 1055 |
|  | 1150 |
|  | 1155 |
|  | 1160 |
|  | 1175 |
| **IBS** | 2770 |
| **CANCER** | 0400 TO 0590 |
| **ALCOHOL PROBLEMS** | 1005* |
|  | 1100* |
|  | 1115 |
| **OTHER PSYCHOACTIVE SUBSTANCE MISUSE** | 1010 |
|  | 1110 |
| **CONSTIPATION** | 2765 |
| **STROKE & TRANSIENT ISCHAEMIC ATTACK** | 2100* |
|  | 2105* |
|  | 2110* |
|  | 2115* |
| **CHRONIC KIDNEY DISEASE** | 2900* |
|  | 2905* |
| **DIVERTICULAR DISEASE OF INTESTINE** | 2760* |
| **ATRIAL FIBRILLATION** | 1990* |
| **PERIPHERAL VASCULAR DISEASE** | 2200* |
|  | 2210 |
|  | 2215* |
|  | 2220* |
|  | 2225* |
|  | 2230* |
| **HEART FAILURE** | 2005* |
|  | 2010* |
|  | 2015* |
|  | 2020* |
| **PROSTATE DISORDERS** | 2960 |
|  | 2970 |
|  | 7960 |
|  | 7965 |
| **GLAUCOMA** | 1535* |
| **EPILEPSY** | 1365 |
|  | 1370 |
|  | 1375 |
| **DEMENTIA** | 1000* |
|  | 1310* |
|  | 4900* |
| **PSYCHOSES** | 1020* |
|  | 1025* |
|  | 1030* |
| **PSORIASIS OR ECZEMA** | 3695 |
|  | 3655 |
|  | 3665 |
| **INFLAMMATORY BOWEL DISEASE** | 2740* |
|  | 2745* |
| **MIGRAINE** | 1380 |
| **BLINDNESS & LOW VISION** | 1555* |
| **CHRONIC SINUSITIS** | 2445 |
| **LEARNING DISABILITY** | 1215* |
|  | 1220* |
| **ANOREXIA OR BULIMIA** | 1170* |
|  | 1180* |
| **BRONCHIECTASIS** | 2505* |
| **PARKINSON’S DISEASE** | 1315* |
|  | 1320* |
| **MULTIPLE SCLEROSIS** | 1335* |
| **VIRAL HEPATITIS** | 0130 |
| **CHRONIC LIVER DISEASE** | 2810* |
|  | 4560* |
| **PAIN** | 1190 |
|  | 3910 |
|  | 3915 |
|  | 3920 |
|  | 3925 |
|  | 3930 |
|  | 3935 |
|  | 3940 |
|  | 3945 |
|  | 3950 |
|  | 3955 |
|  | 3960 |
|  | 3980 |
|  | 3990 |
|  | 3995 |
|  | 4000 |
|  | 4005 |
|  | 4075 |
|  | 4080 |

* If present at one wave it was recorded as present at all subsequent waves, even if not subsequently reported.

Table S- : Twenty-07 baseline and wave 4 sample characteristics by cohort

| **Characteristics** | | **Cohort** | | | | | | | |
| --- | --- | --- | --- | --- | --- | --- | --- | --- | --- |
|  |  | **1970s N (%)** | | | **1950s N (%)** | | **1930s N (%)** | | |
|  |  | Baseline* | Wave 4 | | Baseline | Wave 4 | Baseline | | Wave 4 |
| ***Total*** | | 1,343 | 588 (43.7) | | 1,444 | 788 (54.6) | 1,551 | | 564 (36.4) |
| Multimorbidity | Yes | 35 (2·6) | 106 (18.3) | | 61 (4.2) | 249 (31.6) | 542 (35.0) | | 354 (62.8) |
| Sex | Female | 705 (52·5) | 331 (56.3) | | 788 (54.6) | 437 (55.5) | 849 (54.7) | | 331 (58.7) |
| Age | Mean | 18.6 | 30.1 | | 36.1 | 50.1 | 56.1 | | 69.0 |
| ***Socioeconomic status*** | | | | | | | | | |
| Deprivation | Least | 300 (22.3) | 190 (32.3) | | 372 (25.8) | 305 (38.7) | 278 (17.9) | 169 (30.0) | |
|  | Intermediate | 508 (37.8) | 227 (38.6) | | 601 (41.6) | 298 (37.8) | 574 (37.0) | 209 (37.1) | |
|  | Most | 485 (36.1) | 171 (29.1) | | 471 (32.6) | 185 (23.5) | 699 (45.1) | 186 (33.0) | |
|  | Missing | 50 (3.7) | 0 | | 0 | 0 | 0 | 0 | |
| ***Risk factor*** | | | | | | | | | |
| Smoking | Never | 725 (54·0) | | 257 (43.7) | 547 (37.9) | 304 (38.6) | 475 (30.6) | 207 (36.7) | |
|  | Ex | 156 (11·6) | | 141 (24.0) | 206 (14.3) | 254 (32.2) | 379 (24.4) | 246 (43.6) | |
|  | Current | 460 (34·3) | | 190 (32.3) | 685(47.4) | 230 (29.2) | 697 (44.9) | 111 (19.7) | |
|  | Missing | 2 (0.2) | | 0 | 6 (0·4) | 0 | 0 | 0 | |
| Weekly alcohol unit guidelines | Non/ex-drinker | 123 (9.2) | | 29 (4.9) | 103 (7.1) | 61 (7.7) | 264 (17.0) | 100 (17.7) | |
|  | Current drinker, within limit | 942 (70·1) | | 401 (68.2) | 1,072 (74.2) | 492 (62.4) | 1,086 (70.0) | 387 (68.6) | |
|  | Exceeds limit | 276 (20·6) | | 158 (26.9) | 264 (18.3) | 235 (29.8) | 200 (12.9) | 77 (13.7) | |
|  | Missing | 2 (0·2) | | 0 | 5 (0.4) | 0 | 1 (0.1) | 0 | |
| Diet (fruit or vegetable consumption) | Everyday | 204 (15·2) | | 342 (58.2) | 228 (15.8) | 559 (70.9) | 343 (22.1) | 413 (73.2) | |
|  | Some days | 675 (50·3) | | 201 (34.2) | 713 (49.4) | 189 (24.0) | 786 (50.7) | 129 (22.9) | |
|  | No days | 459 (34·2) | | 45 (7.7) | 342 (23.7) | 40 (5.1) | 336 (21.7) | 22 (3.9) | |
|  | Missing | 5 (0·4) | | 0 | 161 (11.2) | 0 | 86 (5.5) | 0 | |
| Physical activity | 3+ days | 674 (50·2) | | 218 (37.1) | 187 (13.0) | 211 (26.8) | 237 (15.3) | 44 (7.8) | |
|  | 1-3 days | 403 (30·0) | | 159 (27.0) | 392 (27.2) | 166 (21.1) | 284 (18.3) | 85 (15.1) | |
|  | None | 264 (19·7) | | 211 (35.9) | 782 (54.2) | 411 (52.2) | 1,029 (66.3) | 435 (77.1) | |
|  | Missing | 2 (0·2) | | 0 | 83 (5.8) | 0 | 1 (0.1) | 0 | |
| BMI | Healthy | 987 (73.5) | | 255 (43.4) | 772 (53.5) | 233 (29.6) | 638 (41.1) | 155 (27.5) | |
|  | Overweight | 213 (15.9) | | 214 (36.4) | 415 (28.7) | 348 (44.2) | 582 (37.5) | 278 (49.3) | |
|  | Obese | 35 (2.6) | | 81 (13.8) | 101 (7.0) | 151 (19.2) | 160 (10.3) | 106 (18.8) | |
|  | Morbidly obese | 14 (1.0) | | 31 (5.3) | 33 (2.3) | 50 (6.4) | 50 (3.2) | 22 (3.9) | |
|  | Underweight | 74 (5.5) | | 7 (1.2) | 31 (2.2) | 6 (0.8) | 22 (1.4) | 3 (0.5) | |
|  | Missing | 20 (1.5) | | 0 | 92 (6.4) | 0 | 99 (6.4) | 0 | |
| Risk factor count | 0 | 335 (22.4) | | 96 (16.3) | 145 (10.0) | 76 (9.6) | 109 (7.0) | 39 (6.9) | |
|  | 1 | 490 (33.3) | | 217 (36.9) | 389 (26.9) | 240 (30.5) | 429 (27.7) | 150 (26.6) | |
|  | 2 | 352 (23.6) | | 195 (33.2) | 445 (30.8) | 310 (39.3) | 543 (35.0) | 270 (47.9) | |
|  | ≥3 | 141 (9.4) | | 80 (13.6) | 277 (19.2) | 162 (20.6) | 370 (23.9) | 105 (18.6) | |
|  | Missing | 172 (11.4) | | 0 | 188 (13.0) | 0 | 100 (6.4) | 0 | |

* Wave 2 was used for baseline for the 1970s cohort. Data presented are based on complete cases.

N=sample size

Table S- : Number (n) and proportion of sample with each condition, by wave

| **Condition** | **N (%), by wave** | | | | |
| --- | --- | --- | --- | --- | --- |
|  | **1** | **2** | **3** | **4** | **5** |
| HYPERTENSION | 228 (5.1) | 295 (6.5) | 299 (6.6) | 448 (9.9) | 641 (14.2) |
| DEPRESSION | 235 (5.2) | 278 (6.2) | 185 (4.1) | 240 (5.3) | 339 (7.5) |
| RESPIRATORY CONDITIONS including asthma | 200 (4.4) | 199 (4.4) | 196 (4.3) | 212 (4.3) | 246 (5.5) |
| CORONARY HEART DISEASE | 141 (3.1) | 171 (3.8) | 172 (3.8) | 181 (4.0) | 213 (4.7) |
| DYSPEPSIA | 200 (4.4) | 191 (4.2) | 130 (2.9) | 135 (3.0) | 145 (3.2) |
| DIABETES | 41 (0.9) | 48 (1.1) | 64 (1.4) | 99 (2.2) | 156 (3.5) |
| THYROID | 46 (1.0) | 58 (1.3) | 65 (1.4) | 93 (2.1) | 154 (3.4) |
| RHEUMATOID ARTHRITIS, other inflammatory polyarthropathies & systematic connective tissue disorders | 40 (0.9) | 86 (1.9) | 66 (1.5) | 88 (2.0) | 116 (2.6) |
| HEARING PROBLEMS | 72 (1.6) | 184 (4.1) | 193 (4.3) | 236 (5.2) | 284 (6.3) |
| ANXIETY & OTHER NEUROTIC, STRESS-RELATED, & SOMATOFORM DISORDERS | 152 (3.4) | 226 (5.0) | 201 (4.5) | 199 (4.4) | 310 (6.9) |
| IBS | 19 (0.4) | 39 (0.9) | 59 (1.3) | 64 (1.4) | 73 (1.6) |
| CANCER | 29 (0.6) | 37 (0.8) | 44 (0.1) | 58 (1.0) | 93 (2.1) |
| ALCOHOL PROBLEMS | 14 (0.3) | 26 (0.6) | 25 (0.6) | 40 (0.9) | 40 (0.9) |
| OTHER PSYCHOACTIVE SUBSTANCE MISUSE | 3 (0.1) | 3 (0.1) | 5 (0.1) | 5 (0.1) | 7 (0.2) |
| CONSTIPATION | 24 (0.5) | 40 (0.9) | 12 (0.3) | 2 (0.1) | 4 (0.1) |
| STROKE & TRANSIENT ISCHAEMIC ATTACK | 21 (0.5) | 23 (0.5) | 39 (0.9) | 52 (1.2) | 80 (1.8) |
| CHRONIC KIDNEY DISEASE | 3 (0.1) | 4 (0.1) | 7 (0.2) | 4 (0.1) | 17 (0.4) |
| DIVERTICULAR DISEASE OF INTESTINE | 18 (0.4) | 27 (0.6) | 42 (0.9) | 61 (1.4) | 90 (2.0) |
| ATRIAL FIBRILLATION | 2 (0.1) | 2 (0.1) | 4 (0.1) | 11 (0.2) | 19 (0.4) |
| PERIPHERAL VASCULAR DISEASE | 30 (0.7) | 46 (1.0) | 55 (1.2) | 78 (1.7) | 103 (2.3) |
| HEART FAILURE | 11 (0.2) | 12 (0.3) | 10 (0.2) | 13 (0.3) | 25 (0.6) |
| PROSTATE DISORDERS | 13 (0.3) | 28 (0.6) | 34 (0.8) | 42 (0.9) | 52 (1.2) |
| GLAUCOMA | 11 (0.2) | 13 (0.3) | 18 (0.4) | 22 (0.5) | 41 (0.9) |
| EPILEPSY | 37 (0.8) | 39 (0.9) | 32 (0.7) | 29 (0.6) | 27 (0.6) |
| DEMENTIA | 2 (0.0) | 0 (0.0) | 0 (0.0) | 0 (0.0) | 7 (0.2) |
| PSYCHOSES | 9 (0.2) | 10 (0.2) | 11 (0.2) | 15 (0.3) | 24 (0.5) |
| PSORIASIS OR ECZEMA | 127 (2.8) | 169 (3.7) | 149 (3.3) | 216 (4.8) | 226 (5.0) |
| INFLAMMATORY BOWEL DISEASE | 20 (0.4) | 22 (0.5) | 23 (0.5) | 27 (0.6) | 31 (0.7) |
| MIGRAINE | 191 (4.2) | 201 (4.5) | 161 (3.6) | 193 (4.3) | 201 (4.5) |
| BLINDNESS & LOW VISION | 17 (0.4) | 21 (0.5) | 23 (0.5) | 24 (0.5) | 38 (0.8) |
| CHRONIC SINUSITIS | 7 (0.2) | 11 (0.2) | 16 (0.4) | 9 (0.2) | 16 (0.4) |
| LEARNING DISABILITY | 7 (0.2) | 7 (0.2) | 6 (0.1) | 5 (0.1) | 6 (0.1) |
| ANOREXIA OR BULIMIA | 2 (0.0) | 3 (0.1) | 3 (0.1) | 3 (0.1) | 2 (0.1) |
| BRONCHIECTASIS | 3 (0.1) | 2 (0.1) | 2 (0.1) | 3 (0.1) | 3 (0.1) |
| PARKINSON’S DISEASE | 3 (0.1) | 2 (0.1) | 1 (0.1) | 2 (0.1) | 21 (0.5) |
| MULTIPLE SCLEROSIS | 8 (0.2) | 9 (0.2) | 12 (0.3) | 10 (0.2) | 15 (0.3) |
| VIRAL HEPATITIS | 1 (0.1) | 0 (0.0) | 1 (0.1) | 2 (0.1) | 0 (0.0) |
| CHRONIC LIVER DISEASE | 3 (0.1) | 4 (0.1) | 2 (0.1) | 6 (0.1) | 11 (0.2) |
| PAIN | 707 (15.7) | 851 (18.9) | 802 (17.8) | 785 (17.4) | 878 (19.5) |

Table S- : Proportion of sample with each condition by cohort at baseline*

| **Condition** | **N (%), by cohort** | | |
| --- | --- | --- | --- |
|  | **1970s** | **1950s** | **1930s** |
| HYPERTENSION | 1 (0.1) | 16 (1.1) | 212 (13.7) |
| DEPRESSION | 71 (4.7) | 18 (1.2) | 213 (13.7) |
| RESPIRATORY CONDITIONS including asthma | 54 (3.6) | 36 (2.5) | 80 (5.2) |
| CORONARY HEART DISEASE | 0 (0.0) | 4 (0.3) | 136 (8.8) |
| DYSPEPSIA | 6 (0.4) | 48 (3.3) | 145 (9.3) |
| DIABETES | 3 (0.2) | 7 (0.5) | 31 (2.0) |
| THYROID | 3 (0.2) | 8 (0.6) | 36 (2.3) |
| RHEUMATOID ARTHRITIS, other inflammatory polyarthropathies & systematic connective tissue disorders | 0 (0.0) | 10 (0.7) | 28 (1.8) |
| HEARING PROBLEMS | 13 (0.9) | 11 (0.8) | 44 (2.8) |
| ANXIETY & OTHER NEUROTIC, STRESS-RELATED, & SOMATOFORM DISORDERS | 59 (3.9) | 35 (2.4) | 110 (7.1) |
| IBS | 1 (0.1) | 4 (0.3) | 10 (0.6) |
| CANCER | 0 (0.0) | 5 (0.3) | 22 (1.4) |
| ALCOHOL PROBLEMS | 0 (0.0) | 3 (0.2) | 11 (0.7) |
| OTHER PSYCHOACTIVE SUBSTANCE MISUSE | 0 (0.0) | 2 (0.1) | 1 (0.1) |
| CONSTIPATION | 0 (0.0) | 6 (0.4) | 4 (0.3) |
| STROKE & TRANSIENT ISCHAEMIC ATTACK | 1 (0.1) | 2 (0.1) | 18 (1.2) |
| CHRONIC KIDNEY DISEASE | 0 (0.0) | 0 (0.0) | 3 (0.2) |
| DIVERTICULAR DISEASE OF INTESTINE | 0 (0.0) | 1 (0.1) | 17 (1.1) |
| ATRIAL FIBRILLATION | 0 (0.0) | 1 (0.1) | 1 (0.1) |
| PERIPHERAL VASCULAR DISEASE | 3 (0.2) | 2 (0.1) | 26 (1.7) |
| HEART FAILURE | 0 (0.0) | 0 (0.0) | 11 (0.7) |
| PROSTATE DISORDERS | 0 (0.0) | 1 (0.1) | 12 (0.8) |
| GLAUCOMA | 0 (0.0) | 0 (0.0) | 11 (0.7) |
| EPILEPSY | 8 (0.5) | 10 (0.7) | 18 (1.2) |
| DEMENTIA | 0 (0.0) | 1 (0.1) | 0 (0.0) |
| PSYCHOSES | 1 (0.1) | 5 (0.3) | 4 (0.3) |
| PSORIASIS OR ECZEMA | 19 (1.3) | 36 (2.5) | 26 (1.7) |
| INFLAMMATORY BOWEL DISEASE | 4 (0.3) | 7 (0.5) | 10 (0.6) |
| MIGRAINE | 12 (0.8) | 28 (1.9) | 108 (7.0) |
| BLINDNESS & LOW VISION | 2 (0.1) | 7 (0.5) | 7 (0.5) |
| CHRONIC SINUSITIS | 0 (0.0) | 3 (0.2) | 3 (0.2) |
| LEARNING DISABILITY | 6 (0.4) | 1 (0.1) | 0 (0.0) |
| ANOREXIA OR BULIMIA | 1 (0.1) | 0 (0.0) | 0 (0.0) |
| BRONCHIECTASIS | 0 (0.0) | 1 (0.1) | 2 (0.1) |
| PARKINSON’S DISEASE | 0 (0.0) | 0 (0.0) | 3 (0.2) |
| MULTIPLE SCLEROSIS | 0 (0.0) | 1 (0.1) | 7 (0.5) |
| VIRAL HEPATITIS | 0 (0.0) | 0 (0.0) | 0 (0.0) |
| CHRONIC LIVER DISEASE | 0 (0.0) | 0 (0.0) | 2 (0.1) |
| PAIN | 14 (0.9) | 154 (10.7) | 518 (33.4) |

* Baseline is wave 1 for 1950s and 1930s and wave 2 for 1970s cohorts.

Table S- :Test of statistical interaction between sex and cohort on the odds of multimorbidity

|  |  | **Model 1** | |
| --- | --- | --- | --- |
| **Independent variables** |  | **OR** | **95% CI** |
| Sex | Male | 1 |  |
|  | Female | 1.00 | (0.85,1·17) |
| Cohort | 1930 | 1 |  |
|  | 1950 | 1·47 | (1.06,2.05)* |
|  | 1970 | 1.80 | (1.01,3.21)* |
| Age | + 1 year | 1·16 | (1·00,1·34) |
| Multimorbidity at previous wave | No | 1 |  |
|  | Yes | 8·53 | (7·58,9·60)* |
| Quadratic age |  | 1·00 | (0·99,1·00) |
| Cubic age |  | 1·00 | (1·00,1·00)* |
| Sex*Cohort | 1930s female | 1 |  |
|  | 1950s female | 1.24 | (0·99,1·55) |
|  | 1970s female | 1.51 | (1.11,2.05)* |

Analysis based on multiply imputed data. * indicates statistical significance at p<0.05.

Table S- : Summary of missing values for imputed variables

|  | **n observed/imputed** | | | |  |  |
| --- | --- | --- | --- | --- | --- | --- |
|  | **Wave (t-1/t)** | | | |  |  |
| **Variable (at t-1)** | 1/2 | 2/3 | 3/4 | 4/5 | Total observed/imputed | % imputed |
| Smoking | 2995/0 | 3818/102 | 2956/329 | 2649/12 | 12,418/443 | 3.44 |
| Alcohol | 2995/0 | 3818/102 | 2925/360 | 2642/19 | 12,380/481 | 3.74 |
| Physical activity | 2911/84 | 3831/89 | 2887/398 | 2653/8 | 12,282/579 | 4.50 |
| BMI | 2804/191 | 3793/127 | 2848/437 | 2553/108 | 11,998/863 | 6.71 |
| Diet | 2748/247 | 3775/145 | 2882/403 | 2553/108 | 11,958/903 | 7.02 |
| Area-based deprivation | 2995/0 | 3765/155 | 2885/400 | 2569/92 | 12,214/647 | 5.03 |
| Multimorbidity | 2995/0 | 3833/87 | 2972/313 | 2661/0 | 12,461/400 | 3.11 |
| Age | 2995/0 | 3920/0 | 3285/0 | 2661/0 | 12,861/0 | 0.0 |
| Sex | 2995/0 | 3920/0 | 3285/0 | 2661/0 | 12,861/0 | 0.0 |
| Social class* | 2975/20 | 3822/98 | 2972/313 | 2660/1 | 12,429/432 | 3.36 |
| Self-rated health* | 2831/164 | 3826/94 | 2966/319 | 2655/6 | 12,278/583 | 4.53 |
| **Outcome (at t)** |  |  |  |  |  |  |
| Multimorbidity | 2490/505 | 2972/948 | 2661/624 | 2172/489 | 10,295/2,566 | 20.0 |
| **Total n in analysis** | 2995 | 3920 | 3285 | 2661 | 12,861 |  |

* Auxiliary variables

Table S- :Odds of multimorbidity comparing area-based deprivation and household income (n=10,083)

| **Independent variables** |  | **Area-based deprivation and income, adjusted in separate models** | |
| --- | --- | --- | --- |
|  |  | **OR** | **(95% CI)** |
| **Area-based deprivation** | Least | 1 |  |
|  | Intermediate | 1.26 | (1.06,1.49)* |
|  | Most | 1.49 | (1.24,1.79)* |
| **Income** | Highest | 1 |  |
|  | Medium | 1.49 | (1.23,1.81)* |
|  | Lowest | 1.53 | (1.25,1.87)* |
| **Income*sex (ref male) §** | Highest | 1 |  |
|  | Medium | 0.75 | (0.58,0.97)* |
|  | Lowest | 1.02 | (0.79,1.33) |

This table presents equivalent findings to Table 1 (Model 1a) in the main paper, with household income used as an alternative variable to assess socioeconomic status. * indicates statistical significance at p<0.05.

All models are adjusted for age, age^2^, age^3^, sex, cohort, prior multimorbidity, time between waves and sex*cohort interaction. Complete case data were used in nested models.

**§**For income-based analysis, a statistically significant interaction was found between income and sex. The income model was therefore additionally adjusted for and income*sex interaction term.

Table S- :Odds of multimorbidity comparing weekly alcohol with binge drinking (n=8,051)

| **Independent variables** |  | **Weekly alcohol units and daily alcohol units, adjusted in separate models** | |
| --- | --- | --- | --- |
|  |  | **OR** | **(95% CI)** |
| **Alcohol units (recommended maximum weekly units)** | No excess | 1 |  |
|  | Exceeds | 1.05 | (0.90,1.22) |
|  | None | 1.46 | (1.23,1.74)* |
| **Alcohol units (recommended maximum daily units)** § | No excess | 1 |  |
|  | Exceeds | 1.28 | (1.06,1.55)* |
|  | None | 1.21 | (0.88,1.65) |
| **Alcohol units (daily) * sex (ref male)** | Female no excess | 1 |  |
|  | Female exceeds | 0.66 | (0.48,0.91)* |
|  | Female none | 1.35 | (0.93,1.97)* |

This table presents equivalent findings to Table 1 (Model 1c) in the main paper, with daily units of alcohol used as an alternative variable to assess alcohol intake. * indicates statistical significance at p<0.05.

All models are adjusted for age, age^2^, age^3^, sex, cohort, prior multimorbidity, time between waves and sex*cohort interaction. Complete case data were used in nested models.

§For daily alcohol-based analysis, a statistically significant interaction was found between daily alcohol intake and sex. The daily units model was therefore additionally adjusted for daily units*sex.

Table S- :Odds of multimorbidity or death by risk factors & socioeconomic deprivation (n=9,634)

| **Independent variables** |  | **Separate models for each risk factor plus deprivation** | | **Mutually adjusted for all risk factors** | |
| --- | --- | --- | --- | --- | --- |
|  |  | **OR** | **(95% CI)** | **OR** | **(95% CI)** |
| **Area-based deprivation** | Least | 1 |  |  |  |
|  | Intermediate | 1·32 | (1·12, 1·55)* |  |  |
|  | Most | 1·56 | (1·31, 1·86)* |  |  |
| **Smoking** | Never | 1 |  | 1 |  |
|  | Ex | 1·33 | (1·16, 1·51)* | 1·35 | (1·18, 1·54)* |
|  | Current | 1·59 | (1·41, 1·79)* | 1·60 | (1·41, 1·81)* |
| **Alcohol units (recommended weekly units)** | No excess | 1 |  | 1 |  |
|  | Exceeds | 1·15 | (1·00,1·32)* | 1·08 | (0·94,1·24) |
|  | None/Ex | 1·49 | (1·27,1·75)* | 1·49 | (1·27,1·76)* |
| **Diet (fruit or vegetable consumption)** | Everyday | 1 |  | 1 |  |
|  | Some days | 1·15 | (1.02, 1·29)* | 1·10 | (0.98, 1·23) |
|  | No days | 1·59 | (1·35, 1·88)* | 1·45 | (1·23, 1·71)* |
| **Physical activity** | Some | 1 |  | 1 |  |
|  | Little | 0·96 | (0·83, 1·12) | 0·95 | (0·81, 1·11) |
|  | None | 1·05 | (0·92, 1·20) | 1.00 | (0·87, 1·15) |
| **BMI** | Healthy | 1 |  | 1 |  |
|  | Overweight | 1·23 | (1·10, 1·38)* | 1·28 | (1·14, 1·43)** |
|  | Obese | 1·38 | (1·17, 1·62)* | 1·45 | (1·22, 1·71)* |
|  | Morbid obese | 1·92 | (1·46, 2·52)* | 2·00 | (1·52, 2·64)* |
|  | Underweight | 1·56 | (1·02, 2·37)* | 1·36 | (0·88, 2·10) |
| **Risk factor count** | 0 | 1 |  |  |  |
|  | 1 | 1.23 | (1.03, 1.47)* |  |  |
|  | 2 | 1.53 | (1.29, 1.83)* |  |  |
|  | ≥3 | 2.03 | (1.67, 2.47)* |  |  |

This table presents equivalent findings to Table 1 in the main paper, with an alternative classification of the outcome (experiencing either 2+ long-term health conditions or death) to limit potential survivorship bias. * indicates statistical significance at p<0.05.

All models are adjusted for age, age^2^, age^3^, sex, cohort, prior multimorbidity, time between waves and sex*cohort interaction. Complete case data were used.

Table S- :Complete case analysis: odds of multimorbidity by risk factors & socioeconomic deprivation (n=9,277)

| **Independent variables** |  | **Separate models for each risk factor plus deprivation** | | **Mutually adjusted for all risk factors** | |
| --- | --- | --- | --- | --- | --- |
|  |  | **OR** | **(95% CI)** | **OR** | **(95% CI)** |
| **Area-based deprivation** | Least | 1 |  |  |  |
|  | Intermediate | 1·27 | (1·07, 1·50)* |  |  |
|  | Most | 1·48 | (1·23, 1·78)* |  |  |
| **Smoking** | Never | 1 |  | 1 |  |
|  | Ex | 1·31 | (1·14, 1·49)* | 1·33 | (1·16, 1·52)* |
|  | Current | 1·51 | (1·33, 1·70)* | 1·52 | (1·34, 1·72)* |
| **Alcohol units (recommended weekly units)** | No excess | 1 |  | 1 |  |
|  | Exceeds | 1·14 | (0·99,1·32) | 1·08 | (0·93,1·24) |
|  | None/Ex | 1·51 | (1·28,1·78)* | 1·51 | (1·28,1·78)* |
| **Diet (fruit or vegetable consumption)** | Everyday | 1 |  | 1 |  |
|  | Some days | 1·13 | (1.01, 1·27)* | 1·09 | (0.97, 1·23) |
|  | No days | 1·59 | (1·34, 1·88)* | 1·47 | (1·24, 1·74)* |
| **Physical activity** | Some | 1 |  | 1 |  |
|  | Little | 0·96 | (0·82, 1·12) | 0·95 | (0·81, 1·11) |
|  | None | 1·02 | (0·89, 1·17) | 0.98 | (0·85, 1·12) |
| **BMI** | Healthy | 1 |  | 1 |  |
|  | Overweight | 1·27 | (1·13, 1·42)* | 1·31 | (1·16, 1·47)* |
|  | Obese | 1·41 | (1·19, 1·67)* | 1·47 | (1·24, 1·74)* |
|  | Morbid obese | 1·88 | (1·42, 2·49)* | 1·95 | (1·47, 2·57)* |
|  | Underweight | 1·43 | (0·92, 2·24) | 1·27 | (0·80, 2·00) |
| **Risk factor count** | 0 | 1 |  |  |  |
|  | 1 | 1.21 | (1.01, 1.45)* |  |  |
|  | 2 | 1.48 | (1.24, 1.77)* |  |  |
|  | ≥3 | 1.93 | (1.58, 2.36)* |  |  |

This table presents equivalent findings to Table 1 in the main paper, with data from complete cases used rather than multiply imputed data. * indicates statistical significance at p<0.05.

All models are adjusted for age, age^2^, age^3^, sex, cohort, prior multimorbidity, time between waves and sex*cohort interaction.

Table S- : Sensitivity analysis: odds of multimorbidity (3+ conditions) by risk factors & socioeconomic deprivation (n=9,277)

| **Independent variables** |  | **Model 1a-g: separate models for each risk factor plus deprivation** | | **Model 2: mutually adjusted for all risk factors** | |
| --- | --- | --- | --- | --- | --- |
|  |  | **OR** | **(95% CI)** | **OR** | **(95% CI)** |
| **Area-based deprivation** | Least | 1 |  |  |  |
|  | Intermediate | 1·58 | (1·17, 2·15)* |  |  |
|  | Most | 2·04 | (1·46, 2·84)* |  |  |
| **Smoking** | Never | 1 |  | 1 |  |
|  | Ex | 1·48 | (1·18, 1·85)* | 1·50 | (1·20,1·87)* |
|  | Current | 1·92 | (1·55, 2.38)* | 1·94 | (1·56,2·41)* |
| **Alcohol units (recommended weekly units)** | No excess | 1 |  | 1 |  |
|  | Exceeds | 1·38 | (1.11,1·73)* | 1·30 | (1·04,1·63) |
|  | None/Ex | 1·88 | (1·48,2·40)* | 1·88 | (1·48,2·39)* |
| **Diet (fruit or vegetable consumption)** | Everyday | 1 |  | 1 |  |
|  | Some days | 1·18 | (0.99, 1·41) | 1·11 | (0·93,1·33) |
|  | No days | 1·72 | (1·33, 2·23)* | 1·50 | (1·15,1·94)* |
| **Physical activity** | Some | 1 |  | 1 |  |
|  | Little | 1.22 | (0·96, 1·56) | 1·21 | (0·94,1·54) |
|  | None | 1·28 | (1·03, 1·59)* | 1·23 | (0·99,1·52) |
| **BMI** | Healthy | 1 |  | 1 |  |
|  | Overweight | 1·52 | (1·26, 1·83)* | 1·61 | (1·34,1·94)* |
|  | Obese | 1·56 | (1·21, 2·02)* | 1·69 | (1·31,2·18)* |
|  | Morbid obese | 3.29 | (2·23, 4·84)* | 3·61 | (2·45,5·33)* |
|  | Underweight | 3.01 | (1·60, 5·68)* | 2·58 | (1·35,4·92)* |
| **Risk factor count** | 0 | 1 |  |  |  |
|  | 1 | 1.48 | (1.09, 2.02)* |  |  |
|  | 2 | 1.90 | (1.39, 2.60)* |  |  |
|  | ≥3 | 2.82 | (2.01, 3.95)* |  |  |

This table presents equivalent findings to Table 1 in the main paper, with an alternative classification of the outcome (experiencing 3+ long-term health conditions). * indicates statistical significance at p<0.05.

All models are adjusted for age, age^2^, age^3^, sex, cohort, prior multimorbidity, time between waves and sex*cohort interaction. Complete case data were used.

Table S- :Relative indices of inequality (RII) in the development of multimorbidity (3+ conditions), calculated by area-level deprivation, n=9,277 *

|  | **RII Odds Ratio (95% CI)** | **% attenuation^+^** |
| --- | --- | --- |
| Null model | 2.52 (1.78,3.57) | NA |
| Plus smoking | 2.23 (1.58,3.15) | 13.4 |
| Plus diet | 2.36 (1.66,3.34) | 7.4 |
| Plus physical activity | 2.51 (1.77,3.56) | 0.5 |
| Plus alcohol | 2.42 (1.71,3.42) | 4.5 |
| Plus BMI | 2.33 (1.64,3.30) | 8.6 |
| Plus all five risk factors | 1·86 (1.32,2.62) | 33.7 |
| Plus risk factor count | 2.18 (1.54,3.08) | 13.6 |

This table presents equivalent findings to Table 2 in the main paper, with an alternative outcome variable (multimorbidity calculated as 3+ conditions rather than 2+ conditions).

Adjusted for prior multimorbidity, age, age^2^, age^3^, sex, cohort, time between waves and sex*cohort interaction.

^+^ 100*(βModel 2a – βModel 2a + risk factor) / βModel 2

Due to the hierarchical nature of the data and the use of multiple imputation, it was not possible to use Monte Carlo simulation to estimate 95% CI.

Table S- : Relative indices of inequality (RII) in the development of multimorbidity (2+ conditions), calculated by area-level deprivation using the KHB causal mediation approach, n=9,277

|  | **Odds ratio** | **95% CI** | **p value** | **% mediated** | **% mediated in**  **main analysis** |
| --- | --- | --- | --- | --- | --- |
| **Smoking** |  |  |  | 15.2 | 15.4 |
| Total effect | 1.68 | 1.40-2.01 | <0.001 |  |  |
| Direct effect | 1.55 | 1.29-1.86 | <0.001 |  |  |
| Indirect effect | 1.08 | 0.58-2.01 | 0.803 |  |  |
| **Diet** |  |  |  | 11.1 | 11.9 |
| Total effect | 1.68 | 1.40-2.01 | <0.001 |  |  |
| Direct effect | 1.59 | 1.32-1.90 | <0.001 |  |  |
| Indirect effect | 1.06 | 0.67-1.68 | 0.806 |  |  |
| **Alcohol** |  |  |  | 6.6 | 5.3 |
| Total effect | 1.68 | 1.40-2.01 | <0.001 |  |  |
| Direct effect | 1.62 | 1.35-1.94 | <0.001 |  |  |
| Indirect effect | 1.03 | 0.67-1.61 | 0.879 |  |  |
| **Physical activity** |  |  |  | 1.0 | 1.1 |
| Total effect | 1.68 | 1.41-2.01 | <0.001 |  |  |
| Direct effect | 1.67 | 1.40-2.00 | <0.001 |  |  |
| Indirect effect | 1.01 | 0.93-1.09 | 0.9 |  |  |
| **BMI** |  |  |  | 7.5 | 8.3 |
| Total effect | 1.68 | 1.41-2.01 | <0.001 |  |  |
| Direct effect | 1.62 | 1.35-1.94 | <0.001 |  |  |
| Indirect effect | 1.04 | 0.60-1.81 | 0.89 |  |  |
| **Risk factor count** |  |  |  | 18.0 | 17.8 |
| Total effect | 1.68 | 1.40-2.01 | <0.001 |  |  |
| Direct effect | 1.53 | 1.28-1.83 | <0.001 |  |  |
| Indirect effect | 1.10 | 0.56-2.15 | 0.787 |  |  |
| **All risk factors** |  |  |  | 40.3 | 40.8 |
| Total effect | 1.67 | 1.40-2.00 | <0.001 |  |  |
| Direct effect | 1.36 | 1.13-1.64 | 0.001 |  |  |
| Indirect effect | 1.23 | 0.43-3.56 | 0.703 |  |  |

The KHB method estimates both direct and indirect effects that can be interpreted under a counterfactual framework of causation. In addition, it overcomes the potential problem of comparison across different logistic regression models that can arise from non-collapsibility of odds ratios by simultaneously estimating both direct and indirect pathways. To account for the lack of independence of observations, the hierarchical nature of the data was accounted for through the use of Stata’s cluster command. The analysis is based on complete cases.

Note that the beta coefficients from the model were used to calculate the percentage mediated, rather than odds ratios (which are shown above to ease comparison).
